# Knowledge, attitude and practice of farmers towards ticks and tick borne diseases in selected districts of west Gojam, Ethiopia: Implications for control and prevention

**DOI:** 10.1016/j.parepi.2026.e00534

**Published:** 2026-08-19

**Authors:** Gedam Tola, Sissay Menkir, Yelfwagash Asmare, Melaku Wale, Abaineh Munshea

**Affiliations:** a Department of Biology, College of Science, Bahir Dar University, P.O. Box 79, Bahir Dar, Ethiopia; b Department of Biology, College of Natural and Computational Science, Debark University, P.O. Box 90, Debark, Ethiopia

**Keywords:** Amhara region, KAP, TBD, Acaricide management

## Abstract

**Background:**

Ticks and tick-borne diseases (TBDs) pose a serious threat to livestock productivity in Ethiopia, which leads to considerable economic losses. Smallholder farmer's knowledge, attitudes, and practices play a major role for effective management.

**Objectives:**

This study aimed to assess the level of knowledge, attitude, and practice (KAP) of farmers regarding ticks and TBDs in the West Gojam zone, Amhara Region, and identify factors determining disease and vector management.

**Methods:**

A community-based cross-sectional study was conducted involving 384 farmers using a multi-stage sampling technique and a structured questionnaire. KAP levels were assessed using a standardized scoring system and classified into binary outcomes. Data were analysed using SPSS version 26; Chi-square tests and binary logistic regression were used to figure out socio-demographic determinants of KAP at a 95% confidence level.

**Results:**

While 59.4% of respondent's demonstrated adequate knowledge and 89.8% maintained a favourable attitude towards tick and TBD control, regarding implementation, 49.22% of participants exhibited good management practices. Logistic regression revealed that awareness levels and specific animal husbandry practices were statistically significant associations with knowledge and attitude (*p* < 0.05). Crucially, while knowledge scores were positively correlated with acaricide usage, higher knowledge did not consistently correspond to effective application.

**Conclusion:**

Farmers in the study area demonstrated moderate knowledge and highly favourable attitudes towards tick control, alongside weaker and more inconsistent reported management practices. Intervention strategies should focus on bridging these practical gaps through targeted veterinary extension services.

## Graphical abstract

**Figure:**
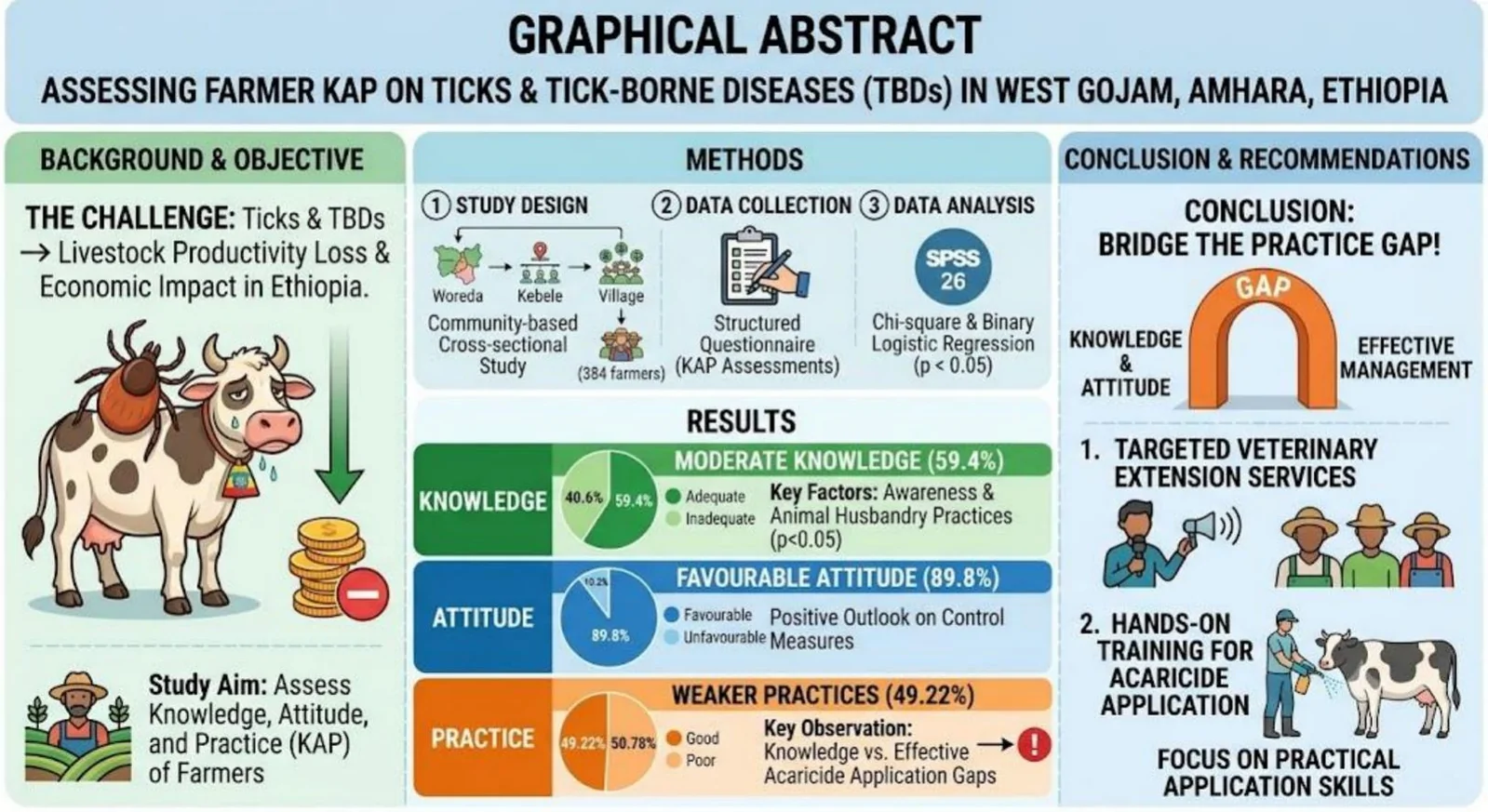

## 1 Introduction

### 1.1 Background of the study

Tick infestations and tick-borne diseases (TBDs) pose a severe threat to global animal health and livestock productivity across diverse agro-ecological environments (Gabriel et al., 2025; Namgyal et al., 2021; Narita et al., 2022). In Ethiopia, where livestock rearing is a cornerstone of rural livelihoods (Alemayehu et al., 2022). Livestock production faces persistent health and management challenges. While major TBDs specifically *Anaplasmosis*, *Babesiosis*, *Theileriosis,* and *Heart water* and diverse tick vectors are widely reported across the country, there is a distinct lack of localized scientific evidence regarding what farmers in the West Gojam zone know, believe, and practice regarding tick management. Understanding this human dimension is key to explaining the on-going disconnect between available control technologies and the continued high prevalence of infestations (Paucar-Quishpe et al., 2024).

While ecological factors dictate tick suitability, the community's daily management habits heavily influence vector persistence and control outcomes on the ground. Investigating local knowledge, attitudes, and practices (KAP) allows for the identification of specific socio-behavioural barriers, knowledge gaps regarding tick ecology, and inadequate control habits that undermine conventional management strategies (Jamra et al., 2024). Furthermore, describing current farmer practices particularly regarding chemical application provides essential context for addressing the emerging threat of localized acaricide resistance.

Characterizing these local misconceptions and practical challenges is necessary for the design of targeted veterinary extension services. By establishing a clear descriptive baseline, this study provides the specific data required to tailor site-appropriate tick control recommendation and extension frameworks suited to the needs of the West Gojam farming community (Narita et al., 2022). While ixodid ticks represent a well-documented threat to cattle productivity across Ethiopia, localized data regarding how smallholder farmers understand and manage these parasites remain scarce. Specifically, in the West Gojam Zone, despite high cattle densities and varying agro-ecological conditions, there has been no systematic assessment of smallholder Knowledge, Attitudes, and Practices (KAP). Understanding these community level factors is crucial for designing locally adaptable tick control strategies. This study aims to assess the current status of KAP regarding tick infestations among cattle owners in West Gojam, identifying key socio-demographic factors associated with these domains without assuming a direct causal link to regional ecological tick distribution.

## 2 Methodology

### 2.1 Study area

**Fig. 1:**
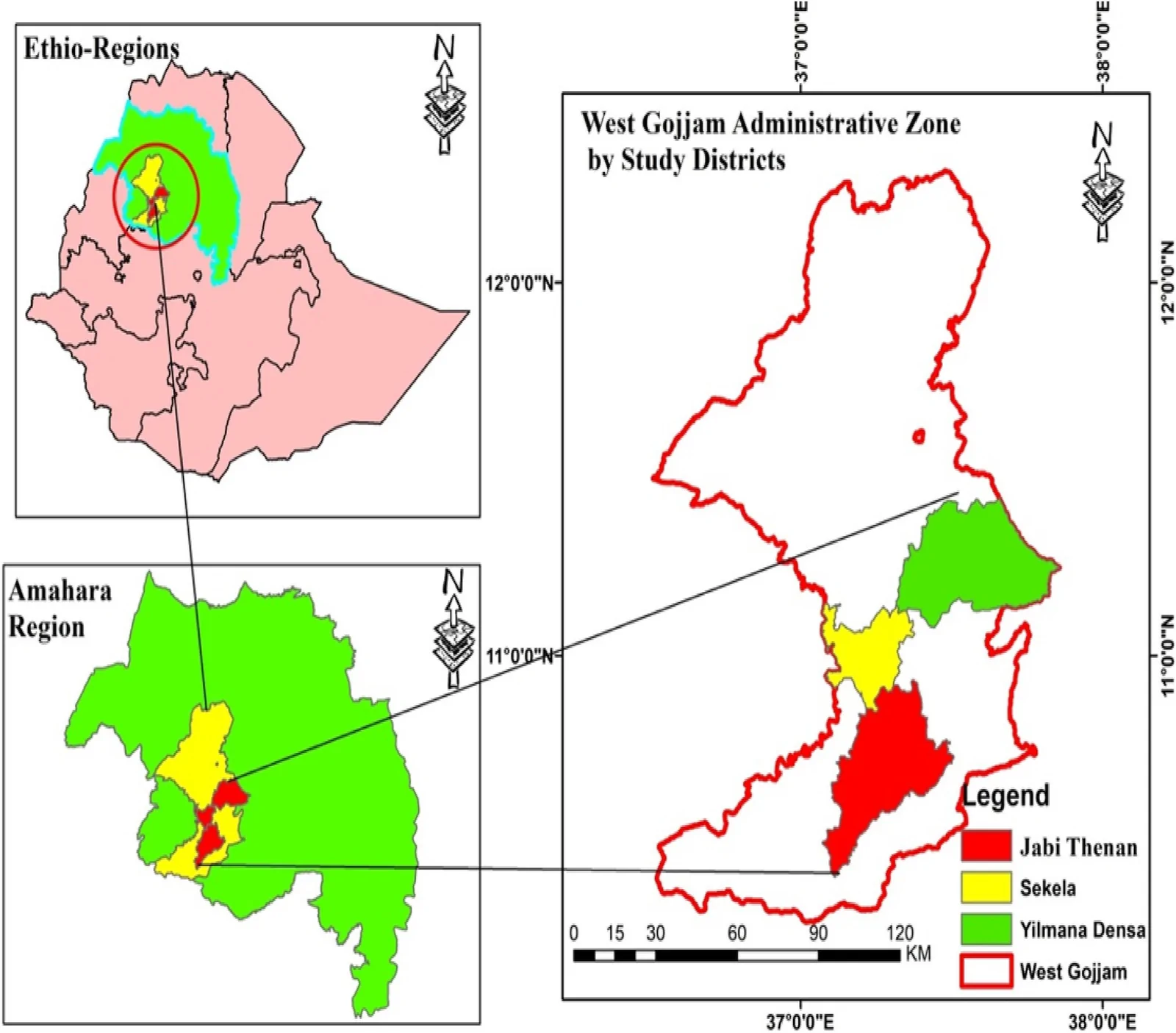
Map of the study area.

The study was conducted in three purposively selected districts; Jabi Tehnan, Yilmana Densa, and Sekela, West Gojam Zone, Amhara region, north-western Ethiopia (Fig. 1). West Gojam is one of the 11 administrative zones in the Amhara Region, comprising 22 districts with an estimated total population of 2,795,775, (Negash et al., 2024). The area is predominantly agrarian, and livestock production plays a central role in farmer's livelihoods. Cattle, sheep, and goats are the most commonly kept livestock species. These districts were selected due to their high cattle population density and the importance of ticks and tick-borne diseases in affecting livestock productivity.

### 2.2 Study design

A community-based cross-sectional study was conducted between February 2023 to May 2025. The study population consisted of household heads who owned livestock in the selected districts. Data were collected using a structured, pre-tested questionnaire designed to capture socio-demographic characteristics and to assess farmers' knowledge, attitudes, and practices (KAP) regarding ticks and tick-borne diseases. The questionnaire also included items related to tick control methods (e.g., acaricide application, manual removal, and use of traditional remedies).

### 2.3 Sampling technique and sample size determination

A multi-stage sampling design was employed. First, the study districts within the West Gojam Zone were purposively selected based on their high livestock populations and veterinary reports of tick prevalence. Second, kebeles were randomly selected from the districts, followed by a simple random selection of householders. Within each district, households owning cattle were considered as the primary sampling units, and participants were proportionally allocated to the districts based on household population size. The sample size of the study was estimated by using sample size estimation formula of n = Z^2^ P (1− P) /e^2^ where: n = required sample size, Z = 1.96 (standard normal deviate at 95% confidence level), *P* = 50%, as there is no similar study in the study area and we assumed 50% of farmers would have adequate KAP about ticks and TBDs, e = margin of error (0.05). Accordingly, a total of 384 households with an additional 10% of sample size included in to account for possible non-response, resulting in a total of 422 farmers expected to participate. However, due to challenges such as household displacement, mobility of some livestock keepers, and occasional unwillingness to participate, data were successfully collected from 384 households.

### 2.4 Data collection

Data were collected through face to face interviews using a pretested structured questionnaire developed in English and translated into the local language (Amharic) to ensure comprehension. The items of questionnaire were adapted from previously validated KAP studies on livestock health (Namgyal et al., 2021), with modifications to fit the local context. The questionnaire consisted of 48 questions divided into the following domains: 1, Socio-demographic (age, gender, marital status, educational status and husbandry practice) 2, Knowledge (18 items): awareness of ticks, identification of tick species, knowledge of tick-borne diseases, and understanding of transmission and prevention. 3, Attitudes (3 items): perceptions of the importance of tick control and beliefs about acaricide use.4, Practices (22 items): current tick control methods, acaricide application and management practice, use of traditional remedies, and treatment history.

The questionnaire was pre-tested on 20 farmers in the Bahir Dar area to check clarity, appropriateness, and reliability; based on the feedback obtained from the pre-test necessary adjustments were made before full deployment. Trained enumerators conducted face-to-face household interviews, filling in responses based on farmers' answers. Prior to each interview, enumerators explained the objectives of the study and obtained verbal informed consent. Respondents were assured of confidentiality, voluntary participation, and their right to withdraw at any stage of the interview without any condition and that their responses would be kept confidential.

### 2.5 Scoring value

As a questionnaire consists of a set of items related to knowledge, attitude, and practice, the respondent's answers to these items were scored based on correctness and accuracy. The full set of 18 knowledge and 22 practice questions included exploratory and descriptive items that do not carry a “correct” or “incorrect” value. Scoring was restricted to the 7 core binary items in each domain that directly measures actionable, evidence-based management. The scores were calculated by assigning a numerical value to each response option and summing up the scores for all the items and the mean value calculated and assigned by cutting value. The resulting score represents the level of knowledge, attitude, and practice of the respondents as described in Supplementary Table S1and S2.

The method used by Tack et al. (2013) and Namgyal et al. (2021) was adapted to categorize the respondents as either having “adequate knowledge” or “inadequate knowledge”. Of the total 18 knowledge related questions, scoring was done to seven questions with binary response if they answer >3 questions correctly and mean score, >0.43, was taken as they have adequate Knowledge in other way considered as having inadequate knowledge when the respondent's answer ≤3 questions and the mean score between (0–0.43).

Likewise, the attitude of the respondents was assessed using a Likert scale from three items to determine overall score respondent's opinions towards tick prevention and control programs and absolute modified Bloom's criterion of 70% (cut-off score of 3.41) used to classify as having favourable attitude for mean value score > 3.41 and unfavourable attitude for mean score value <3.41.Finally, To evaluate the level of practice among respondents, the continuous mean practice score (ranging from 0 to 2) was categorized into binary levels: ‘Poor Practice’ and ‘Good Practice’. Following standard KAP study conventions, the theoretical midpoint of 1.00 was utilized as the cut-off value. Participants scoring below 1.00 were classified as having poor practice, while those scoring 1.00 or higher were classified as having good practice.

### 2.6 Statistical analysis

The collected data were cleaned, coded and validated for completeness into Excel before exporting to SPSS version 26 for analysis and Quantum GIS Software version 10.7 were used for making study area maps. Descriptive statistics (frequency and percentage) were used to summarize socio demographic characteristics and the full dataset response was subjected to a descriptive analysis.

A Chi-square test (χ^2^) was used to assess the association between categorical variables, with statistical significance set at 95% level of significance. To identify factors associated with KAP levels a bivariate and multivariate logistic regression model were performed. The variable selected purposefully from previous literature and based on clinical relevance the final binary logistic regression model was constructed using a forward stepwise selection method. A likelihood ratio test was utilized to determine the significant variables that improved the likelihood ratio statistic the most. The strength of the association were determined by the odds ratio (OR) with 95% confidence interval (CI).Confounding was determined by combining the variables excluded from the final model (Chowdhury and Turin, 2020).

Various statistical techniques were used to assess reliability, including correlation analysis for test-retest and inter-rater reliability and Cronbach's Alpha for internal consistency. Cronbach's Alpha produced internal consistency that exceeds the minimum value of 0.70 that required for acceptable reliability. The calculated Cronbach's alpha values were 0.710 for the knowledge, 0.708 for the attitude, and 0.734 for the practice, demonstrating acceptable internal reliability across all subscales (Izah et al., 2024)**.** Hosmer-Lemeshow test was considered for a goodness of fit test for logistic regression models to assess whether the predicted probabilities of the outcome match the observed probabilities across different subgroups of the data and when the *p*-value is greater than 0.05 alpha levels, we conclude that the model fits the data well.

## 3 Results

### 3.1 Socio-demographic characteristics of respondents

Of the total 384 farmers participated in this survey from three selected district of West Gojam Zone which consists of different agro ecology, 147(38.3%) were from Yilmana Densa(Woina Dega or Mid-land), 131(34.1%) from Jabi Tehnan(Mid-land) and 106(27.6%) from Sekela district (Dega or High land) of west Gojam zone. Regarding educational level, almost over half of respondents, (200; 52.1%) did not attend any school, while the remaining attended non-formal education (73; 19.0%), primary level (70; 18.2%), and secondary level and above were (41; 10.68%). The majority of the respondents were male, (256, 66.7%), while female constituted (128, 33.3%) of the study population. Most participants were above 45 years old (199, 51.9%) followed by those in age group of 36–45 (110, 28.6%) while majority of participants were married (318, 82.8%). Of the total 384 farmers, majority, 230(59.9%), rear local cattle breeds and mainly 94 (88.68%) from Sekela followed by Yilmana Densa and Jabi Tehnan. Cross breed cattle were mainly reared by farmers from Jabi thenan followed by those from Yilmana Densa and Sekela. Regarding the husbandry practice, majority of the respondents, apply a mix of stall feeding, free and tethered grazing. Farmers from Yilmana Densa districts mostly follow this practice 82 (55.78%) followed by Sekela 71 (66.98%) and Jabithenan districts 62 (47.32%). The practices of all time free grazing and stall feeding were more applicable among farmers in Jabithenan 30 (22.90%), 39 (29.77%) respectively than others (Table 1).

**Table 1:** Socio-demographic characteristics of the respondents in three selected district of West Gojam zone.

Variable	Category	Jabi Tehnan (*n* = 131(34.1%)	Sekela (*n* = 106 (27.6%)	Yilmana Densa (*n* = 147(38.3%)	Total (*N* = 384) and %
					
Age(in year)	18–35	19(14.50%)	23(21.70%)	33(22.45%)	75(19.5%)
	36–45	41(31.30%)	24(22.64%)	45(30.61%)	110(28.6%)
	Above 45	71(54.20%)	59(55.66%)	69(46.94%)	199(51.9%)
Educational –level	Not attend any School	74(56.49%)	35(33.02%)	91(61.90%)	200(52.1%)
	Attending non-formal education	23(17.56%)	10(9.43%)	40(27.21%)	73(19.0%)
	Primary school	14(10.69%)	48(45.28%)	8(5.44%)	70(18.2%)
	Secondary school and above	20 (15.27%)	13(12.26%)	8 (5.44%)	41 (10.68%)
Gender	Female	58(44.27%)	41(38.68%)	29(19.73%)	128(33.3%)
	Male	73(55.73%)	65(61.32%)	118(80.27%)	256(66.7%)
Kebeles	Adet	0(0.00%)	0(0.00%)	51(34.69%)	51(13.3%)
	Bahirzafa	0(0.00%)	28(26.42%)	0(0.00%)	28(7.3%)
	Finote selam	45(34.35%)	0(0.00%)	0(0.00%)	45(11.7%)
	Gumbla	0(0.00%)	44(41.51%)	0(0.00%)	44(11.5)
	Jiga	36(27.48%)	0(0.00%)	0(0.00%)	36(9.4%)
	Konch	0(0.00%)	0(0.00%)	38(25.85%)	38(9.9%)
	Mankusa	50(38.17%)	0(0.00%)	0(0.00%)	50(13.0%)
	Walka	0(0.00%)	0(0.00%)	58(39.46%)	58(15.1%)
	Werayita	0(0.00%)	34(32.08%)	0(0.00%)	34(8.9%)
Marital Status	Divorced	7(5.34%)	1(0.94%)	24(16.33%)	32(8.3%)
	Married	104(79.39%)	102(96.23%)	112(76.19%)	318(82.8%)
	Single	20(15.27%)	3(2.83%)	11(7.48%)	34(8.9%)
Cattle breed they have	Cross	56 (42.75%)	9 (8.49%)	41 (27.89%)	106(27.6%)
	Exotic	25 (19.08%)	3 (2.83%)	20 (13.61%)	48(12.5%)
	Local	50 (38.17%)	94 (88.68%)	86 (58.50%)	230(59.9%)
Husbandry practice	All time free grazing	30 (22.90%)	22 (20.75%)	2 (1.36%)	54(14.1%)
	Mix of stall feeding,free and tethered grazing	62 (47.32%)	71 (66.98%)	82 (55.78%)	215 (55.99%)
	Stall feeding	39 (29.77%)	13 (12.26%)	63 (42.86%)	115(29.9)

### 3.2 Socio-demographic characteristics and overall KAP level

The overall assessment of knowledge, attitude, and practice (KAP) levels revealed that the majority of 277(72.1%) the respondents had moderate KAP levels, while 16.1% (*n* = 62) and 11.7% (*n* = 45) showed good and poor KAP levels, respectively. There was statistical significant association between KAP level and the variables, gender, district and husbandry practice (*p* < 0.02, <0.001, and *p* < 0.001) respectively while age category, educational status and marital status did not show any significant association (Table 2).

**Table 2:** Socio-demographic Characteristics with KAP level.

Variables	Total (N)384	KAP level			
		Good n (%) 62(16.1%)	Moderate n (%) 277(72.1%)	Poor n (%) 45(11.7%)	*P* value
					
Gender					<0.02*
Male	256	34(13.28%)	196(76.56%)	26(10.15%)	
Female	128	28(21.87%)	81(63.28)	19(14.84%)	
Age Category					0.169
>45	199	29(14.57%)	153(76.8%)	17(8.5%)	
36–45	110	22(20%)	72(65.45%)	16(14.54%)	
18–35	75	11(14.66%)	52(69.33%)	12(16.00%)	
Districts					<0.001*
Jabi Tehnan	131	24(18.32%)	100(76.33%)	7(5.34%)	
Sekela	106	11(10.37%)	68(64.15%)	27(25.47%)	
Yilmana Densa	147	27(18.36%)	109(74.14%)	11(7.48%)	
Educational status					0.726
Attending non- formal education	73	14(19.17%)	54(73.97%)	5(6.84%)	
Not attend any School	200	25(12.5%)	150(75%)	25(12.5%)	
Primary school	70	12(17.14%)	50(71.42%)	8(11.42%)	
Secondary school and above	41	11 (26.83%)	23 (56.10%)	7(17.07%)	
Marital status					0.576
Divorced	32	6(18.75%)	24(75%)	2(6.25%)	
Married	318	49(15.40%)	228(71.69%)	41(12.89%)	
Not married	34	7(20.58%)	25(73.52%)	2(5.88%)	
Husbandry Practice					<0.001*
All time free grazing	54	10(18.51%)	39(72.2%)	5(9.25%)	
Mix of stall feeding, tethered grazing and free grazing	215	31(14.42%)	149(69.30%)	35(16.28%)	
Stall feeding	115	21(18.26%)	89(77.39%)	5(4.34%)	

Specifically, a higher percentage of female respondents had Good KAP compared to male respondents. Participants from Sekela district had the highest incidence of Poor KAP, in contrast to those from Jabi Tehnan and Yilmana Densa. In terms of husbandry practice, participants who practiced stall feeding had the best KAP results (77.39% moderate, 18.26% good), while those who implemented a mix of stall feeding, free grazing and tethered grazing who had the most cases with poor KAP (24.74%) outcomes. Husbandry practices were significantly associated with KAP levels (*p* < 0.001). Respondents who practiced stalls feeding demonstrated better outcomes, achieving moderate (77.39%) and good (18.26%) KAP levels. Conversely, participants who practiced mixed feeding approach had the highest prevalence of poor KAP as compared to for free grazing and for exclusive stall feeders.

### 3.3 Assessment of Association of Knowledge Attitude and practice of farmers towards tick and tick born disease

#### 3.3.1 Knowledge of farmers towards tick and tick born disease among districts

**Fig. 2:**
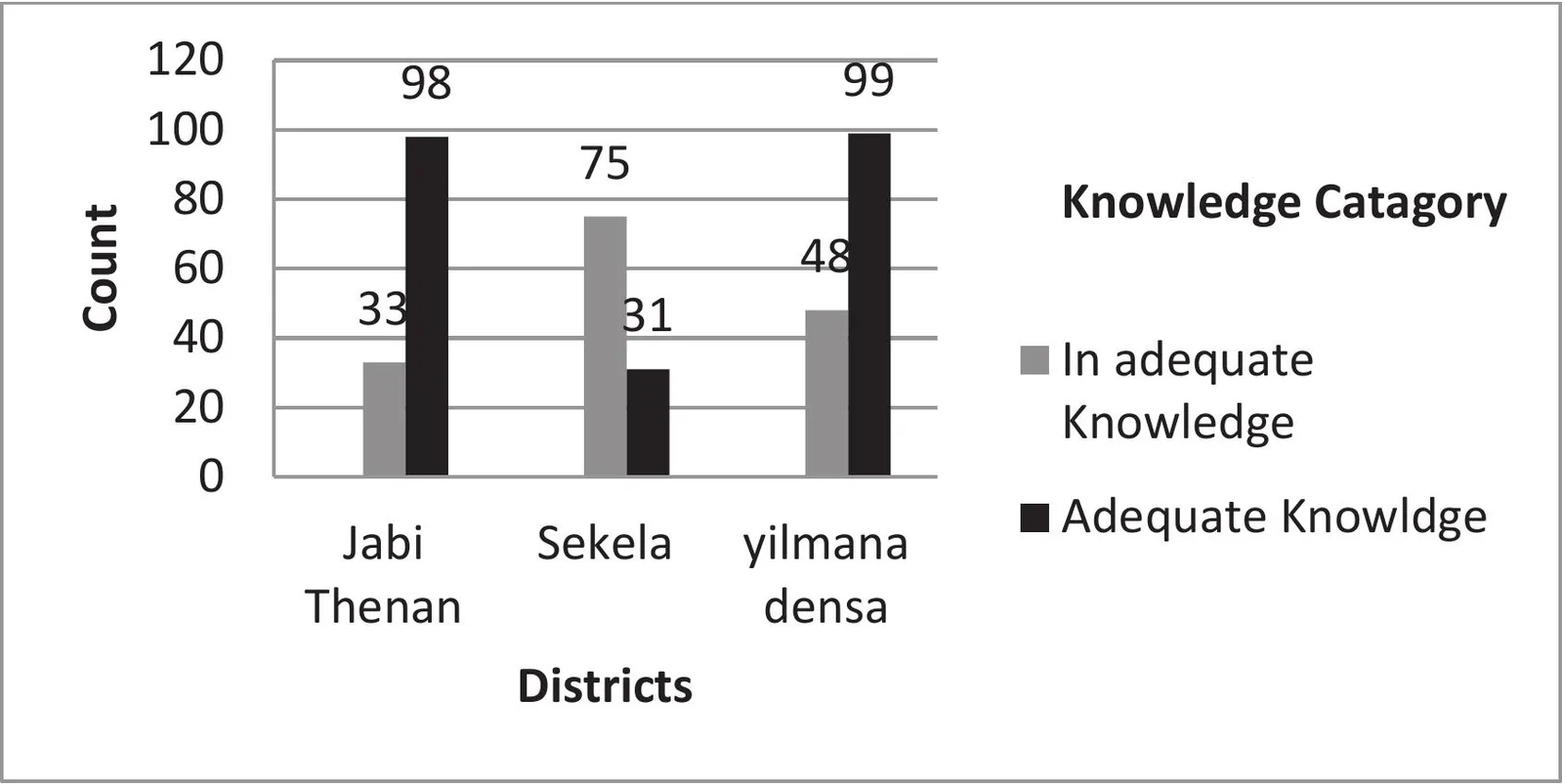
Knowledge of the farmers towards ticks and TBD within the district.

The result revealed that Knowledge of farmers towards ticks and tick-borne disease varied within districts, higher proportion of farmers from Jabi Tehnan and Yilmana Densa had adequate knowledge as compared to those in Sekela district (Figs. 2).

Most respondents reported that they have seen ticks on the animal's body; 80(20.8%) in the forests; 10(2.6%) in grazing land and the rest 95(24.5%) reported they have seen ticks in all locations, in grazing land, on the animal's body and in the forest. The majority, 163(42.4%), of the respondents, were not aware of how cattle become infested with ticks, while 103 (26.8%) identified the forest as the source of infestation, and 80(20.8%), 5(1.3%), 33(8.6%) identified grazing land, bedding materials and fodder grasses, as the other sources of tick infestation respectively. A total of 244 respondents (63.5%) reported that the ticks are commonly seen in summer (June, July, and August), while 65(16.9%) reported having seen them in winter (December, January, and February). Whereas, the majority of the respondents reported that ticks can be available throughout the year. One hundred and fifty-eight respondents indicated that ticks are commonly found in warm places,while 10 reported ticks can survive in cold places. However, 124 (32.2%), reported ticks can be found in both warm and cold places. Regarding susceptibility of breed to ticks, the majority of the respondents 200 (52.1%) believed that the local breeds are more susceptible to tick infestation, 126 (32.8%) participants believed that exotic breeds are more susceptible, 25(6.5%) said that both local and exotic breeds are infested by ticks whereas the remaining 33 (8.6%) of the study subjects responded that they “Don't know”.

Most of the respondents 195(50.8%) responded that old cattle are more susceptible to tick infestation whereas 62(16.1%), 19(4.9%), 53(13.8%) of them replied that adults, heifers, young calves, are more infested respectively while 16 (4.2%) of the respondents said “Don't Know”. Still other 39(10.2%) responded that cattle of all ages are vulnerable to tick infestation. When farmers were asked where they heard about tick borne disease (TBD), the majority 313(81.5%) replied that they did not hear about TBD before whereas, the remaining 71 (18.5%) indicated that they heard about TBDs before. Among those (71) who heard about TBD before, 14(19.7%), 31(43.7%) and 26(36.6%) heard from farmers training program, neighbours and friends respectively. When farmers were asked about their level of understanding towards how human can get tick born disease, majority 204(53.1%) of the respondents replied that they don't know and the remaining 180(46.9%) respondents reported that they believe ticks transmit disease to humans Supplementary Table S3.

#### 3.3.2 Association of Knowledge's with demographic characteristics towards tick and TBD

As shown in Table 3 of the total of 384 respondents, 228 (59.4%) of them had adequate knowledge and the remaining 156 (40.6%) had inadequate knowledge towards tick and TBD. Chi-square(*X*^*2*^) analyses revealed that districts where farmers reside, animal species they own and their husbandry practice of the farmers are significantly associated with their level of knowledge. A higher proportion of respondents from Jabi Tehnan 90(68.70%) had adequate knowledge as compared to farmers those live in Yilmana Densa 87(59.18%) and Sekela 51 (48.11%) (*X*^*2*^ = 10.300 and P value = 0.006.Respondents who own exotic (68.75%) and cross breed (70.75%) cattle had much higher adequate knowledge than those who own local cattle (52.17%) cattle breed type (*X*^*2*^ = 12.384 and *p* = 0.002*). This result may suggest that rearing of the improved cattle breed likely to increase their knowledge as those breed need special management. There was statistically significant association between husbandry practices of famers with their statuses of knowledge towards ticks and TBDs (X^2^ = 16.618 *p* = 0.001). All time free grazers (75.93%) had more adequate knowledge than those who implement stall feeding (67.83%) and mix of stall feeding, free and tethered grazing (50.70%). While age, marital status, gender and education level were not significantly associated with famers.

**Table 3:** Association of Knowledge's with demographic characteristics towards tick and TBD in west Gojam.

Variables Category		Inadequate Knowledge (156 (40.6%))	Adequate Knowledge (228 (59.4%))	Total (*N* = 384)	Chi square (*X*^*2*^) value	P value
Districts	Jabi Tehnan	41(31.30%)	90(68.70%)	131	10.300	0.006*
	Sekela	55(51.89%)	51(48.11%)	106		
	Yilmana Densa	60(40.82%)	87(59.18%)	147		
Gender	Male	101(39.45%)	155(60.55%)	256	0.437	0.508
	Female	55(42.97%)	73(57.03%)	128		
Age	>45	82(41.21%)	117(58.79%)	199	0.058	0.972
	18–35	30(40.00%)	45(60.00%)	75		
	36–45	44(40.00%)	66(60.00%)	110		
Educational status	Not attend any School	81(40.50%)	119(59.50%)	200	2.035	0.565
	Attending non-formal education	32(43.84%)	41(56.16%)	73		
	Primary school	24(34.29%)	46(65.71%)	70		
	Secondary school and above	19(46.34%)	22(53.66%)	41		
Marital Status	Divorced	13(40.63%)	19(59.38%)	32	0.089	0.956
	Married	130(40.88%)	188(59.12%)	318		
	Single	13(38.24%)	21(61.76%)	34		
Animal species they have	Local	110(47.83%)	120(52.17%)	230	12.384	0.002*
	Exotic	15(31.25%)	33(68.75%)	48		
	Cross	31(29.25%)	75(70.75%)	106		
Husbandry practice	Stall feeding	37(32.17%)	78(67.83%)	115	16.618	0.001*
	Mix of stall feeding, free and tethered grazing	106(49.30%	109(50.70%)	215		
	All time free grazing	13(24.07%)	41(75.93%)	54		

Knowledge status towards ticks and TBDs.

#### 3.3.3 Attitudes of farmers towards tick and TBD

Likert scale measurement showed that majority 205 (53.4%) of the respondents strongly agree that proper use of acaricide can reduce the incidences of tick infestation in cattle. In addition, about half 192(50%) and 180(46.9%) of respondents mentioned that they strongly agree that the risk of the tick infestation can be reduced by keeping the cattle in the shade and adopting good farm practices (like regular cleaning of floor, regular checking of animals body, avoiding the use of bedding materials) respectively. Generally, the mean score of all items is above 4, which indicates that respondents generally agree that appliying accaricide, keeping cattle in the shade and adopting good farming practice (GFP) can reduce tick infestation as described in Table 4.

**Table 4:** Scale measurement of farmer's attitude regarding acaricide usage, resting cattle in a shade, and GFPs in relation to risk of tick infestation and TBDs.

Variables	Disagree		Neutral		Agree		Strongly Agree		Descriptive statistics		
	N	%	N	%	N	%	N	%	Mean	Median	S D
											
Use of Acaricides can reduce Tick.	40	10.4%	20	5.2%	119	31.0%	205	53.4%	4.27	5.00	0.964
Resting cattle in a Shade can reduce risk of tick infestation.	20	5.2%	41	10.7%	131	34.1%	192	50.0%	4.29	4.50	0.856
GFP_can reduce risk of tick and TBDs.	27	7.0%	29	7.6%	148	38.5%	180	46.9%	4.25	4.00	0.874

(GFP = good farming practice)

While assessing the relationship among items of attitude for internal consistency with correlation analysis as shown in Table 5, it was revealed that attitude of the farmers about the use of acaricide was not related to the resting cattle in the shade as a control measure of tick and TBD. As Pearson correlation coefficient (*r* = s 0.015), which indicated weak, positive correlation, and almost no linear relationship with Sig. (2-tailed) *p* value of 0.774, which implies the correlation was not statistically significant (*p* > 0.01).

**Table 5:** Pearson Correlation for attitude internal consistency of variables.

Variables		Use of acaricide can reduce tick infestation	Resting cattle in a shade can reduce tick and TBDs	GFP reduce risk of tick infestation and TBDs
			
Use of acaricides can reduce tick infestation	Pearson Correlation	1		
	Sig. (2-tailed)			
	N	384		
Resting cattle in a Shade can reduce tick and TBDs	Pearson Correlation	0.015	1	
	Sig. (2-tailed)	0.774		
	N	384	384	
GFP reduce risk of tick infestation and TBDs	Pearson Correlation	0.063	0.202^⁎⁎^	1
	Sig. (2-tailed)	0.215	0.000	
	N	384	384	384

** Correlation is significant at the 0.01 level (2-tailed), N: The sample size, which is 384 for all comparisons.

In the same way, the attitude of using acaricide was not related to the attitude of applying GFP as a control measure. Pearson correlation coefficient (*r* = 0.063) indicated a very weak positive correlation with *p* > 0.05, suggesting a minimal linear relationship that was not statistically significant.

However, there was a statistically significant, positive association between the attitude that resting cattle in a shade reduces tick and TBDs and the attitude that good farming Practices (GFP) reduces tick and TBDs.

The Pearson correlation coefficient (*r* = 0.202) indicated a weak-to-moderate positive correlation, suggesting that as agreement with one practice increases, agreement with the other tends to increase. The *p*-value (*p* < 0.01; *Sig*. (2-tailed)) indicated that this correlation was highly statistically significant. This finding suggests that these two non-chemical management methods tend to be recognized or accepted together by respondents.

#### 3.3.4 Association of Attitudes with demographic characteristics towards tick and TBD

Of the total of 384 respondents interviewed, majority 345(89.8%) of farmers had favourable attitude, while the remaining 39 (10.2%) had unfavourable attitude towards tick and TBD. Attitude varied slightly across districts, among 147 respondents from Yilmana Densa the majority, 134 (91.16%) of the farmers have favourable attitude, and followed by Jabi Tehnan 118 off 131 (90.08%) and Sekela 93 off 106 (87.74%). Regarding gender as shown in the Table 6 from 256 male respondents 232(90.62%) and from 128 female respondents 113(88.28%) have favourable attitude. Regarding age, respondents whose age was ranged between 36 and 45 and above 45 were relatively less than respondents with age category between 18 and 35 to have favourable attitude 91.82%, 87.94% and 92% respectively and regarding the animal species they have majority 204(88.70%) of respondents who rear the local cattle have favourable attitude than respondents who rear cross and exotics respectively 97(91.51%), 44(91.67%). Association of attitudes with demographic characteristics towards tick and TBD was favourable regardless of the variables included. Out of the many variables involved, including educational status had not statistical significant association (*χ*^*2*^ = 1.627, *p* = 0.653) (Table 6).

**Table 6:** Association of Attitudes with demographic characteristics towards tick and TBD in west Gojam Zone.

Variables Category		Un Favourable Attitude (39(10.2%))	Favourable attitude (345 (89.8%))	Total (N) (384(100%))	Chi square(*X*^*2*^) value	*p* value
					
Districts	Jabi Tehnan	13(9.92%)	118(90.08%)	131	0.802	0.670
	Sekela	13(12.26%)	93(87.74%)	106		
	Yilmana Densa	13(8.84%)	134(91.16%)	147		
Gender	Male	27(10.55%)	229(89.45%)	256	0.128	0.720
	Female	12(9.38%)	116(90.63%)	128		
Age	>45	19(9.55%)	180(90.45%)	199	1.047	0.592
	18–35	10(13.33%)	65(86.67%)	75		
	36–45	10(9.09%)	100(90.91%)	110		
Educational status	Attending non-formal education	5(6.85%)	68(93.15%)	73	1.627	0.653
	Not attend any School	20(10%)	180(90%)	200		
	Primary level	9(12.86%)	61(87.14%)	70		
	Secondary school and above	5(12.20%)	36 (87.80%)	41		
Marital Status	Divorced	5(15.63%)	27(84.38%)	32	1.732	0.421
	Married	32(10.06%)	286(89.94%)	318		
	Single	2(5.88%)	32(94.12%)	34		
Husbandry Practice	Stall feeding	9(7.83%)	106(92.17%	115	1.852	0.604
	Mix of stall feeding,free and tethered grazing	25(11.63%)	190(88.37%)	215		
	All time free grazing	5(9.26%)	49(90.74%)	54		
Cattle breed they own	Local	26(11.30%)	204(88.70%)	230	0.829	0.661
	Exotic	4(8.33%)	44(91.67%)	48		
	Cross	9(8.49%)	97(91.51%)	106		

#### 3.3.5 The practice of the respondent towards tick and TBD

Most farmers 198(51.6%) in the west Gojam zone rear cattle mainly to generate incomes through the sale of products (milk, butter, cheese, meat, etc) followed by those who rear the cattle to use their products for family consumption purposes 102(26.6%), selling animals 62(16.1%) and 22(5.7%) of them use the animals as the source of manure.

The assessment of overall applicability of the husbandry practices at study area indicated that most farmers, 118(30.7%), practised a mix of stall feeding and tethered grazing followed by 115 (29.7%) those who practiced stall feeding alone, 97 (25.3) of farmers who practiced a mix of stall feeding and free grazing and 54 (14.1%) of the farmers practised free grazing throughout the year. With regard to the frequency of cleaning cattle shed floor, most of the farmers 265(69.0%) practised cleaning shed floor on daily basis while the a few, 13(3.4%), never cleaned and left it as it is. About half,185(48.2%), of farmers did not use bedding material for their cattle, however, relatively higher number of the farmers used bedding materials 199(51.8%) for their cattle in the shade. Most, 185 (48.2%), of the farmers in the study area did not use bedding material in their cattle shade. Among those who used the bedding material in the shade, majority, 98(25.5%), used gravel and sand followed by those who used litter leaves 82(21.4%), and straw19(4.9%). Likewise, the time of using the bedding material was diffrent by season. Most of them used it in winter161(41.9%), 21(5.5%), throughout the year, 17(4.4%) during summer wheras 185(48.2%) of farmers did not report use of any bedding material at all.

**Fig. 3:**
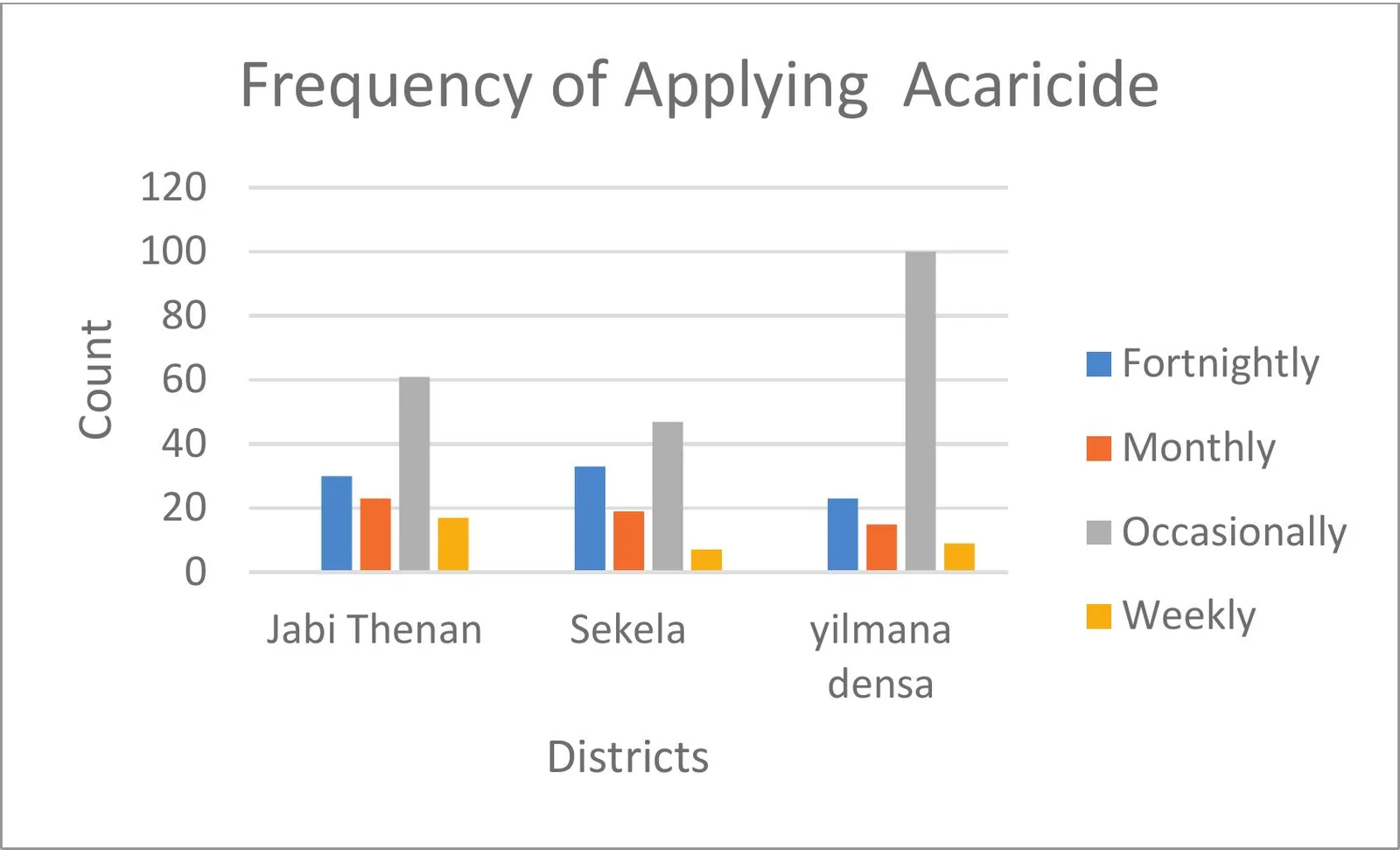
Frequency of Applying Acaricide within District.

When farmers were asked about the control measures they implement to manage tick infestation, majority of them replied that they inject ivermectin 142(37.0%), followed by those who use other acaricides 118(30.7%), adopt good farming practices 43(11.2%), use homemade remedies 38(9.9%), manually remove the ticks 32(8.3%), and 11(2.9%) of them used both acaricides and adopt good farming practices (Table S4).Variation was also observed with the regard to the frequency of applying acaricide on the cattle body. Most of the farmers, 208(54.2%), applied acaricides occasionally, while 33(8.6%) applied on weekly basis, 86(22.4%) fortnightly, and the remaining 57(14.8%) of monthly (Fig. 3).

About half of the farmers 192(50%) had never followed the labeled or enclosed instruction and ignored crucial information on dosage, target ticks, safety, and withdrawal periods, while only 82(21.4%) farmers followed instruction consistently. In the same way, only a small number 85(22.1%) of the farmers were implementing the correct dilution of acaricide while most 167(43.5%) of them did not employ standard measurements, which may lead to tick resistance or toxicity if it applied under and over dosing. Safety wise,159(41.1%) of farmers exposed to toxic chemicals as they were not wearing protective gears at all, whereas nearly a quater 86(22.4%) of the farmers keept their saftey by using appropriate methods. Regarding how the farmers applied acaricide on cattle bodies, 176(45.8%) of the farmers did it correctly, while 139(36.2%) of them did it incorrectly and the remaining 69(18.0%) applied it partialy. Of the total 384 respondents, 133 (34.6%) reported that they never observe the withdrawal period of acaricide, while 82 (21.4%) reported that they always observe it. And about 105(27.3%) of farmers failed to check their body after a direct contact with tick infested cattle. Likewise, 110 (28.6%) of them failed to check their body after environmental exposure (Table S5).

#### 3.3.6 Association of Practice with demographic characteristics towards tick and TBD

The overall score practice of farmers towards acaricide application and personal safety revealed that majority 195(50.78%) of respondents had poor and the remaining 189(49.22%) had good practice towards controlling tick and acaricide management practices(Table 7).There was some variation among districts with regard to farmers practice score, more than half of the farmers from Yilmana Densa 81(55.10%) and Sekela 57(53.77%) had good practice than those who reside in Jabi Thenan 51(38.93%) and there was significant association of practice score and the district where farmers live (*X*^2^ = 8.463 *p* = 0.015).Regarding educational status the respondents,who did not attend any school had poor practice score 118(59.00%) than that of respondents who attended non formal, primary, secondary and above level and education level was significantly associated with practice score (*X*^***2***^ = 11.961 *p* = 0.008). Practice score was also significantly associated with the type of cattle bread they rear (*X*^*2*^ = 7.152,*P* = 0.028); of total 230 farmers who had local cattle breed majority 126(54.78%) of them had good practice and of 106 cross cattle breeders, 44 (41.51%) had good practice while 62(58.49%) had poor practice score and from 48 exotic cattle owner, 19 (39.58%) of them had good practice and the remaining 29(60.42%) of them had poor practice. While age, gender, marital status and husbandary practice were not significantly associated with practice (Table 7).

**Table 7:** Association of practice with demographic characteristics towards tick and TBD in west Gojam Zone.

Variables	Category	Poor practice 195(50.78%)	Good practice 189(49.22%)	Total(N)384	Chi square(*X*^*2*^) value	*p* value
						
Districts	Jabi Tehnan	80 (61.07%)	51(38.93%)	131	8.463	0.015*
	Sekela	49(46.23%)	57(53.77%)	106		
	Yilmana Densa	66(44.90%)	81(55.10%)	147		
Gender	Female	61(47.66%)	67(52.34%)	128	0.750	0.386
	Male	134(52.34%)	122(47.66%)	256		
Age	>45	97(48.74%)	102(51.26%)	199	0.734	0.693
	18–35	39(52.00%)	36(48.00%)	75		
	36–45	59(53.64%)	51(46.36%)	110		
Educational status	Attending non-formal education	29(39.73%)	44(60.27%)	73	11.961	0.008*
	Not attend any School	118(59.00%)	82(41.00%)	200		
	Primary level	32(45.71%)	38(54.29%)	70		
	Secondary school and above	16 (39.02%)	25 (60.98%)	41		
Marital Status	Divorced	16(50.00%)	16(50.00%)	32	0.390	0.823
	Married	160(50.31%)	158(49.69%)	318		
	Single	19(55.88%)	15(44.12%)	34		
Husbandry Practice	Stall feeding	57(49.57%)	58(50.43%)	115	7.332	0.062
	Mix of stall feeding and free and tethered grazing	103(47.91%)	112(52.09%)	215		
	All time free grazing	35(64.81%)	19(35.19%)	54		
Cattle breed they have	Local	104(45.22%)	126(54.78%)	230	7.152	0.028*
	Exotic	29(60.42%)	19(39.58%)	48		
	Cross	62(58.49%)	44(41.51%)	106		

Overall, a variety of concerns involving cattle managing were noticed throughout the observation period, included feeding practices, disposal of waste, domestic management, and the frequency of applying acaricide.

### 3.4 Logistic regression analysis for factors influencing farmers knowledge, attitude and practice

Model goodness-of-fit was evaluated using the Hosmer–Lemeshow test across all three multivariable logistic regression models. All models demonstrated good fit to the empirical data: Knowledge (χ 2 = 10.152,df = 8,*p* = 0.254), Attitude (χ 2 = 6.128,df = 8,*p* = 0.633), and Practice (χ 2 = 9.746,df = 8,*p* = 0.283). All models yielded non-significant *p*-values (*p* > 0.05), confirming good model fit and calibration across all KAP domains.

#### 3.4.1 Factors associated with farmers knowledge

Multivariate analysis revealed that explanatory variables like husbandry practice and attitude were significantly associated with knowledge. Farmers who practiced all time free grazing and stall feeding were 4 times and 2 times more likely, respectively, to have adequate knowledge than those who practiced mix of stall feeding, tethered and free grazing with AOR of 4.662(1.933–11.242) and 2.003(95% CI, 1.162–3.451). Regarding the association of knowledge and attitude, the respondents who had favourable attitude were 11 times more likely to have adequate knowledge than respondents who had un- favourable attitude (AOR =11.202, *p* < 0.05) (Table 8).

**Table 8:** Factors associated with Knowledge (Adequate towards in adequate) of farmers with socio-demographic Factors.

Variables	Bivariate (COR) (95% CI)	*P* value	Multivariate AOR (95% CI)	*p* value
Age				
>45	Ref	0.972		0.527
18–35	1.051(0.612–1.807)	0.856	1.301(0.703–2.407)	0.402
36–45	1.051(0.654–1.690)	0.836	0.880(0.514–1.508)	0.642
District				
Yilmana Densa	Ref	0.006*		0.083
Jabi Tehnan	1.514(0.923–2.482)	0.100	1.354(0.732–2.503)	0.334
Sekela	0.639 (0.387–1.058)	0.082	0.586(0.278–1.233)	0.159
Gender				
Male	Ref			0.509
Female	0.865(0.562–1.330)		0.891(0.528–1.504)	0.665
Marital status				
Married	Ref	0.956		0.674
Divorced	0.905(0.337–2.430)	0.843	1.100(0.456–2.655)	0.832
Single	0.895(0.433–1.852)	0.765	0.704(0.306–1.617)	0.408
Education level				
Not attending any school	Ref			
Attending non formal education	0.872(0.507‐–1.499)	0.618	0.893 (0.483–1.651)	0.720
Primary school	1.305(0.739–2.304)	0.365	1.876 (0.770–4.566)	0.166
Secondary school and above	0.788 (0.396–1.569)	0.501	1.053 (0.419–2.646)	0.912
Animal Species they have				
Local	Ref			0.142
Cross	2.218(1.356–3.627)	0.002*	1.782(0.993–3.196)	0.053
Exotic	2.017(1.039–3.913)	0.038*	1.417 (0.656–3.062)	0.375
Husbandry practice				
Mix of stall feeding and tethered and free grazing	Ref	0.000*		0.000*
All time free grazing	0.326(0.165–0.643)	0.001*	4.662(1.933–11.242)	0.001*
Stall feeding	0.668 (0.320 -1.396)	0.284	2.003(1.162–3.451)	0.012*
Attitude				
Favourable	12.430(4.738–32.604)	0.0001*	11.202(4.083–30.731)	0.000*
Un favourable	Ref			
Practice				
Good practice	0.701(0.465–1.054)	0.088	0.760 (0.476–1.215)	0.252
Poor practice	Ref			

* Statistically significant at p < 0.05; COR = Crude Odds Ratio; AOR = Adjusted Odds Ratio; CI = Confidence Interval; Ref = Reference category.

#### 3.4.2 Factors associated with attitude of farmers

In the bivariate analysis, only educational status (specifically secondary school attendance) demonstrated a statistically significant association with the outcome (*p* = 0.035).The educational level showed a significant relationship with attitude. Compared to those with no formal schooling, participants with a primary school education were nearly four times more likely to have a favourable attitude (AOR = 3.911, 95% CI: 1.010–15.145, *p* = 0.048). In contrast, individuals who attained a secondary school level were approximately 71.6% less likely to have a favourable attitude than those without formal education (AOR = 0.284, 95% CI: 0.104–0.778, *p* = 0.014). Other socio-demographic factors, including age, district of residence, gender, and marital status were not statistical significantly associated with attitude (*p* > 0.05) (Table 9).

**Table 9:** Association of Attitude with Different Factors.

Variables	Category	Bivariate COR (95% CI)	*p* value	Multivariate AOR (95% CI)	*p* value
					
Age	>45	(ref)	0.596	(ref)	0.503
	18–35	0.686 (0.303–1.552)	0.366	0.606 (0.256–1.433)	0.254
	36–45	1.056 (0.472–2.358)	0.895	0.944 (0.409–2.179)	0.893
District	Yilmana Densa	(ref)	0.672	(ref)	0.374
	Jabi Tehnan	0.881(0.393–1.975)	0.758	0.683 (0.273–1.711)	0.416
	Sekela	0.694 (0.308–1.565)	0.379	0.468 (0.160–1.367)	0.165
Gender	Male	(ref)		(ref)	
	Female	1.140(0.557–2.332)	0.720	1.458 (0.6603.224)	0.351
Marital status	Married	Ref	0.432		0.295
	Divorced	0.604 (0.217–1.679)	0.334	0.420 (0.130–1.354)	0.146
	Single	1.790 (0.410–7.821)	0.439	1.383 (0.290–6.585)	0.684
Education	Not attending any school	(ref)	0.035⁎	(ref)	0.018⁎
	Attending non formal education	2.968 (0.949–9.281)	0.920	1.062(0.416–2.713)	0.900
	Primary school	1.994 (0.815–4.881)	0.153	3.911(1.010–15.145)	0.048⁎
	Secondary school and above	4.850 (1.250–18.820)	0.022⁎	0.312 (0.115–0.845)	0.022⁎
Animal Species they have	Local	(ref)	0.662	(ref)	0.728
	Cross	1.374 (0.620–3.044)	0.434	1.441 (0.578–3.591)	0.433
	Exotic	1.402 (0.466–4.220)	0.548	1.234 (0.375–4.065)	0.729
Husbandry practice	Mix of stall feeding and tethered and free grazing	(ref)	0.541	(ref)	0.176
	All time free grazing	1.289 (0.470–3.541)	0.622	2.760 (0.801–9.513)	0.108
	Stall feeding	1.550 (0.698–3.442)	0.282	1.755 (0.723–4.260)	0.214

⁎ Statistically significant at p < 0.05; COR = Crude Odds Ratio; AOR = Adjusted Odds Ratio; CI = Confidence Interval; Ref = Reference category.

#### 3.4.3 Factors associated with practice of farmers

Multivariate analysis showed that Hosmer-Lemeshow test confirmed adequate model goodness-of-fit and the likelihood of good practice among famers in Sekela district was 51.5% lower than farmers in Jabi Tehnan with (AOR =0.515 and 95% CI (0.293–0.902, *p* = 0.020).With the regard to educational status farmers who attained primary, secondary and non-formal education level were more likely to have good practice than those with no educational background (AOR of 1.778, 2.351 and 2.100) respectively. All the other factors like, husbandry practice, age, gender, marital status, and animal breed type they own were not statistically significant in multivariate logistic regression analyses (Table 10).

**Table 10:** Multivariate Logistic Regression and associated factors practice.

Variables	Category	Bivariate (COR) (95% CI)	*p* value	Multivariate AOR (95% CI)	*p* value
Age	>45	(ref)	0.693	1.00 (ref)	0.544
	18–35	0.878(0.516–1.494)	0.631	0.801(0.458–1.402)	0.438
	36–45	0.822 (0.516–1.311)	0.410	0.779 (0.475–1.279	0.324
District	Yilmana Densa	Ref	0.672		0.067
	Sekela	0.881(0.393–1.975)	0.758	0.515 (0.293–0.902)	0.020⁎
	Jabi Tehnan	0.694 (0.308–1.565)	0.379	0.716 (0.362–1.415)	0.337
Gender	Male	Ref	0.453		
	Female	1.206 (0.789–1.845)	0.387	1.402 (0.871–2.258)	0.165
Marital status	Married	Ref	0.824		0.881
	Divorced	1.013 (0.489–2.095)	0.973	0.88 (0.394–1.992)	0.770
	Single	0.799 (0.392–1.629)	0.538	0.839 (0.384–1.832)	0.660
Education	Not attending any school	Ref	0.043⁎		0.034⁎
	Attending non formal education	2.18 (1.264–3.773)	0.005⁎	2.100 (1.180–3.739)	0.012⁎
	Primary school	1.709 (0.988–2.957)	0.055	1.778 (0.941–3.359)	0.076
	Secondary school	1.785 (0.920–3.462)	0.086	2.351(1.069–5.169)	0.033⁎
Animal Species they have	Local	Ref	0.029		0.186
	Cross	0.586 (0.368–0.933)	0.024⁎	0.640 (0.378–1.081)	0.095
	Exotic	0.541 (0.287–1.020)	0.057	0.647 (0.324–1.293)	0.218
Husbandry practice	Mix of stall feeding and tethered and free grazing	Ref	0.086		0.452
	All time free grazing	0.499 (0.269–0.927)	0.028⁎	0.664 (0.327–1.348)	0.257
	Stall feeding	0.936 (0.595–1.472)	0.774	1.070 (0.649–1.764)	0.791

⁎ Statistically significant at p < 0.05; COR = Crude Odds Ratio; AOR = Adjusted Odds Ratio; CI = Confidence Interval; Ref = Reference category.

#### 3.4.4 Correlation between knowledge, attitude and practice score

Correlation analysis of KAP scores showed that the overall KAP score was strongly and highly significantly correlated with knowledge: *r* = 0.523^∗∗^ (*p* < 0.001), attitude: *r* = 0.523^∗∗^ (*p* < 0.001) and practice: *r* = 0.553^∗∗^ (*p* < 0.001). The overall KAP score is likely a composite measure of these three components. The fact that the practice component has the strongest correlation (*r* = 0.553) suggesting that it contributes slightly more to the overall KAP score than knowledge and attitude (Table 11).

**Table 11:** Pearson Correlation matrix examines the relationships between, a KAP and (Knowledge, Attitude, and Practice) on tick and TBD.

		KAP	Knowledge	Attitude	Practice
KAP	Pearson Correlation	1			
	Sig. (2-tailed)				
	N	384			
Knowledge	Pearson Correlation	0.523^⁎⁎^	1		
	Sig. (2-tailed)	0.000			
	N	384	384		
Attitude	Pearson Correlation	0.523^⁎⁎^	0.227^⁎⁎^	1	
	Sig. (2-tailed)	0.000	0.000		
	N	384	384	384	
Practice	Pearson Correlation	0.553^⁎⁎^	0.002	−0.078	1
	Sig. (2-tailed)	0.000	0.969	0.127	
	N	384	384	384	384

** Correlation is significant at the 0.01 level (2-tailed).

Regarding relationships among Knowledge, Attitude, and Practice scores, the person correlation between Knowledge vs. Attitude is 0.227 ^∗∗^ with *p*-value: 0.000, which implies a weak to moderate, but highly significant, positive correlation between knowledge and attitude. This means that individuals with higher knowledge tend to have a more positive attitude, but this relationship is not very strong.

As Pearson correlation coefficient (*r*) between knowledge and practice was 0.002 with *p* value = 0.969, indicating no statistically significant linear relationship between knowledge and practice. This implies that having more knowledge does not predict better practice or behaviour. Likewise, the correlation analysis between attitude and practice is (*r* = −0.078, *p* = 0.127) indicated a very weak, non-significant negative correlation between attitude and practice. Thus, attitude does not significantly predict practice (Table 11).

## 4 Discussion

As this study aimed to assess livestock owner's knowledge, attitude, and practice towards ticks and TBDs along with factors associated with KAP in the West Gojam Zone, Amhara Region, Ethiopia. A critical insight from this study is the apparent disconnect between farmers theoretical understanding and their field-level actions. While a majority of smallholders displayed adequate knowledge (59.4%) and highly favourable attitudes (89.8%), less than half (49.22%) translated this into good management practices. Crucially, higher knowledge scores did not linearly correlate with safer or more effective acaricide application practices. This suggests that holding positive views or recognizing tick risks is insufficient to overcome structural limitations on the ground, such as resource constraints, fluctuating access to high-quality acaricides, or a reliance on traditional, habituated application habits.

In this study, all respondents reported that they had observed ticks, this finding consistent with studies done in Nigeria (Malgwi et al., 2025). This finding might be due to the respondents are farmers whose livelihoods are closely linked to animals, pastures, and forests where ticks are common. Although all respondents had seen ticks, more than half did not know how ticks cause disease and how the cattle became infected. The majority of respondents thought that ticks were seen more frequently during the summer season which is also consistent with the finding of Malgwi et al. (2025). While some of them thought that ticks become more frequently in the winter months and throughout the year. This might be due to most cattle lose their body condition and low immune capacity to build resistance to ticks, during winter (Malgwi et al., 2025; Jamra et al., 2024). Of the total 384 participants in this study, nearly 60% of the respondents had adequate knowledge and about 90% had favourable attitude towards tick and tick-borne disease, this findings align with the findings of (Yizengaw et al., 2026) done in Ethiopia while is lower than the findings from Rwanda and India (Gabriel et al.,2025; Jamra et al., 2024)), and however, the good practice (49.22%) towards tick prevention and control programs is higher than it. Meanwhile attitudes and practices garnered high scores of 90.09% and 90.83% respectively among medical staff in endemic areas of China (Shao et al., 2024) these inconsistencies might be due to the environmental and targeted respondent's variation.

Farmers, who practice mixed feeding approach, were more likely to have adequate knowledge and a favourable attitude than those who implement the stall-feeding system of cattle rearing. Studies done in India mentioned that animals with free grazing were more likely to be infested by ticks than stall-fed ones, and prevalence is lower in the farms where the stall-feeding system is practiced (Jamra et al., 2024). The positive association between the farmers whose husbandry practices were the stall-feeding system and having a favourable attitude can likely be attributed to the adoption of the government's livestock intensification and commercialization programs that promote the stall-feeding system, which most of the farmers in the study area practice. In this system, animals are normally kept inside the sheds and are rarely let out for grazing; this not only protects the animals from accidental falls and fights with other animals but also reduces their exposure to ticks (Ahmed et al., 2020). Consequently, such positive outcomes largely influence the farmers' attitudes. Tick infestation is also known to be determined by the type of housing, breed, and grazing type (Rehman et al., 2017). Our findings revealed a statistically significant association between cattle breed type and farmer knowledge levels. More than half of the respondents recognized exotic breeds of cattle as generally resistant to ticks; some respondents reported a belief that local breeds of cattle are the most infested. This inconsistency in the beliefs could be due to existing differences in the systems of rearing cattle between the exotic and local breeds. Smallholders managing crossbred or improved cattle were more likely to possess adequate knowledge scores compared to those managing indigenous breeds. Rather than implying that breed type directly alters cognitive awareness, this association likely reflects that farmers investing in high-value, improved breeds are more frequently targeted by veterinary extension training or actively seek out management information.

The majority of the respondents mentioned that old and adult cattle are more susceptible to tick infestation than young ones. This result aligns with studies done in Ethiopia by Kemal et al. (2016).The reason for higher tick infestation in the old age cattle groups may be attributed to weak immunity. Moreover, unlike the productive adult cattle that are given the utmost management care, the young calves and old cattle are the least attended. They can, therefore, act as a reservoir for on-going environmental tick contamination (Namgyal et al., 2021).

The result suggested that most of the respondents who used sheds for their cattle in the study area have soil flooring, concrete flooring, and CGI sheet roofing, and daily cleaned floors, and the bedding material used included leaf litter, straw, gravel, and sand. The Likert scale result shows that most of the respondents agree that using a shed can reduce tick infestation. During the winter season, ticks are known to survive well in leaf litter, as it provides consistent insulation from the cold conditions of winter (Linske et al., 2019). Studies in Bhutan (Namgyal et al., 2021) also describe the infestation of cattle in winter can be associated with the use of leaf litter and mention that many farmers in the study area consider leaf litter to be one of the sources of ticks; eventually, in this study, the majority of them did not use it for bedding material. This practice could mitigate the infestation of sheds, but we need to undertake additional studies to ascertain this.

Factors for most poor control practice often identified where, improper use of acaricide (weak solutions, wrong frequency, or failure to treat all animals) directly results in high survival rates and persistent infestation when the respondents use manual tick removal methods, they tend to leave the ticks on the ground rather than collect, burn and flush them with running water and other control practices. This practice might bring about the recycling of tick infestations. Most respondents reported using acaricide on cattle as well as considered it to be the primary method of controlling ticks and reported using acaricide occasionally (whenever there is high tick infestation). This is similar to findings from Ethiopia and Pakistan (Namgyal et al., 2021; Tesfaye and Abate, 2023).Additionally, farmers in South Africa treat tick-infested cattle with acaricide every two weeks during the summer and winter months of the year (Githaka et al., 2022). These findings are indicative of lacking information on the optimal use of acaricide and effective control strategies. Although farmers are advised to strictly adhere to a specific dilution rate, there is no system to monitor the usage in the field and the method of application of acaricide. Effective tick control approaches such as seasonal treatments at the peak of tick activity and intensive treatments at the beginning of the tick season are not followed. Application of acaricide weekly, or every two weeks during the tick propagation season (typically the rainy season in tropical and subtropical Africa), is frequently employed in areas where tick resistance is prevalent (Githaka et al., 2022).So, regular monitoring is an essential part in delaying the development of resistance, as it has been recommended by veterinarians that acaricide treatments should not exceed more than five per season (Abbas et al., 2014).

While this study provides valuable insights into the KAP of farmers in West Gojam, several limitations were encountered. First, the use of a cross-sectional design allows for the identification of associations but precludes the establishment of definitive causal relationships between variables. Second, because the data were self-reported, the results may be subject to social desirability bias, where farmers report practices they perceive as correct, or recall bias regarding historical acaricide usage and disease frequency. Third, the study focused exclusively on the human dimension of tick management and did not include longitudinal biological sampling or laboratory-based acaricide resistance testing to validate the farmer's perceptions of treatment failure. Fourth, although the sample size was robust, the findings are geographically specific to the West Gojam Zone; therefore, the generalizability of the results to other regions with different agro-ecological conditions and livestock husbandry systems should be approached with caution in addition classification of KAP levels relies on arbitrary scoring cut-offs, which introduces inherent categorization uncertainty. Lastly, while a multi-stage sampling strategy was employed to ensure broad representative coverage across the study areas, the binary and multivariate logistic regression models were executed using standard statistical procedures. Consequently, intra-cluster correlations were not explicitly modelled, which may result in slightly narrower confidence intervals. Nevertheless, given our robust sample size and careful sampling frame, the observed odds ratios and overall epidemiological patterns remain highly informative and valid for the study population.

## 5 Conclusion

In conclusion, this study demonstrates that smallholder cattle owners in the West Gojam Zone possess moderate baseline knowledge (59.4%) and highly favourable attitudes (89.8%) regarding tick management, yet less than half (49.22%) consistently implement good tick control practices. These findings justify the development of targeted veterinary extension programs and hands-on training frameworks designed specifically to bridge this implementation gap. Rather than broad theoretical messaging, future public and veterinary health initiatives should prioritize practical, farm-level demonstrations of safe acaricide application strategies.

Overall this study assessed the levels of awareness, attitude, and practice gaps among farmers in relation to tick infestation prevention and control and strategies for ensuring sustainable livestock production and for preventing the cattle from developing tick infestation and TBD as well as acaricide resistance.

The findings demonstrate a notable variation between what farmers know and what they implement in the field, as higher levels of knowledge and favourable attitudes were observed, which did not proportionally result in good management practices. Instead, the study revealed a greater prevalence of poor practices, characterized by the widespread misuse of acaricide, inaccurate dilution methods, and a failure to follow safety protocols. These results suggest that awareness alone is insufficient for disease control, and future interventions are needed to address the socio-economic barriers that prevent farmers from implementing their knowledge, their perceptions, their willingness to participate in control programs, current tick control methods used by farmers, and gaps between practices and observed behaviors.

This study identified that attitude and husbandry practices are determinants of knowledge. However, the persistence of poor practices despite adequate knowledge points towards significant structural and socio-economic barriers, including a lack of access to high-quality equipment and educational resources. Furthermore, the improper application of acaricide might lead to a serious risk for the acceleration of acaricide resistance and treatment failure, threatening the long-term sustainability of livestock production in the study area. So shifting the extension strategy; from knowledge-based awareness campaigns to practice-based facilitation to provide shared, high-quality spraying equipment and centralized, supervised dilution stations to enforce correct concentration, appropriate frequency of application of acaricide, and safety protocols, thereby addressing the economic and infrastructural barriers.

This study also observed that the farmers in the study area perceived ticks and TBDs as significant problems for livestock health. Overall, the findings from this study contribute to better understanding of the farmer's perspectives about ticks and TBDs and also provide useful baseline data for future researchers to carry out further studies. Moreover, it provides information that would be essential for the development and implementation of appropriate policies.

Further research integrating epidemiology and socio-economic impacts of ticks and TBDs is also needed to support a One Health approach, enabling better understanding of disease distribution, improved control strategies, and reduced livestock losses.

## Ethical consideration and consent to participate

Ethical consideration was done in accordance with the Declaration of Helsinki. Ethical approval letter was obtained from Institutional Review Board of Bahir Dar University, Science College (Ref; PRCSVD/672/2023). Support letter was obtained from science collage department of Biology for selected districts animal health office and permission was also obtained from village leaders. Prior to data collection, the study's objectives were explained to each participant in their local language (Amharic), and formal verbal informed consent was obtained from all individual respondents before administering the questionnaire.

## CRediT authorship contribution statement

**Gedam Tola:** Writing – review & editing, Writing – original draft, Software, Methodology, Investigation, Formal analysis, Data curation, Conceptualization. **Sissay Menkir:** Writing – review & editing, Validation, Supervision, Funding acquisition, Conceptualization. **Yelfwagash Asmare:** Visualization. **Melaku Wale:** Visualization. **Abaineh Munshea:** Writing – review & editing, Visualization, Validation, Supervision, Conceptualization.

## Consent for publication

Not applicable.

## Funding source

This study was funded by the Bahir Dar University College of Sciences and funder has no a specific role in the conceptualization, design, data collection, analysis, decision to publish, or preparation of the manuscript.

## Declaration of competing interest

The authors declare that they have no known competing financial interests or personal relationships that could have appeared to influence the work reported in this paper.
