# Supplementary material for: Knowledge, attitude and practice of farmers towards ticks and tick borne diseases in selected districts of west Gojam, Ethiopia: Implications for control and prevention
Source: Parasite Epidemiol Control. 2026 Aug 19;34:e00534. doi: 10.1016/j.parepi.2026.e00534 (PMC13543954; doi:10.1016/j.parepi.2026.e00534)
Supplement: Supplementary file 1 [file mmc1.docx]

# Supplementary material

Table S1.Scored Items and Scoring value for each KAP domain

| **Domain** | **Items** | **Response Options** | **Scoring Assignment** |
| --- | --- | --- | --- |
| ***Knowledge*** | *Item K1: Have you seen a tick?* | Yes / No / | Yes =1  No=0 |
|  | *Item K2: Do you think ticks always stay on the body of cattle unless removed?* | Yes / No /Don't Know | Yes =1  No=0 |
|  | *Item K3: Have you also seen ticks on the body of other animals?* | Yes / No | Yes =1  No=0 |
|  | *Item K4: Do you think cattle can get diseases from ticks?* | Yes / No | Yes =1  No=0 |
|  | *Item K5: Have you heard of any tick-borne diseases in cattle?* | Yes / No | Yes =1  No=0 |
|  | *Item K6: Have you been bitten by ticks?* | Yes / No | Yes =1  No=0 |
|  | *Item K7: Do you think humans can get diseases from the tick bites?* | Yes / No | Yes =1  No=0 |
| **Attitude** | *Item A1: Proper use of synthetic acaricides can reduce the incidences of tick infestation in cattle.* | 5-point Likert Scale | Strongly dis agree=1  Dis agree=2  Neutral=3  Agree=4  Strongly agree=5 |
|  | *Item A2: Risk of the tick infestation can be reduced by keeping the cattle always in the shed* | 5-point Likert Scale | Strongly Disagree = 1 to Strongly Agree = 5 |
|  | *Item A3: Adopting good farm practices can reduce the risk of tick infestation (e.g., regular washing of floor, regular checking of animals, avoiding the use of bedding materials, etc.)* | 5-point Likert Scale | Strongly Disagree = 1 to Strongly Agree = 5 |
| **Practice** | *Item P1:* Do you check your body for ticks after handling the tick-infested cattle? | Always / Sometimes / Never | Never = 0 , Sometimes = 1 Always = 2 |
|  | *Item P2:* Do you check your body for ticks after visiting the forests? | Always / Sometimes / Never | Never = 0 Sometimes = 1 Always = 2 |
|  | *Item P3:* How do you apply the acaricide? |  | Wrong method=0  Partial spraying=1  Spraying correctly=2 |
|  | *Item P4:* Do you read the acaricide label instructions before use? |  | Don't measure=0  By estimation=1  According to label instructions=2 |
|  | *Item P5:* Do you wear protective gear during application? |  | No protection=0  Partial gear =1  Full gear=2 |
|  | *Item P6:*  *How do you determine the acaricide concentration?* |  | Don't measure=0  By estimation=1  According to label instructions=2 |
|  | *Item P7:* Do you observe the withdrawal period after application? |  | Never =0  Sometimes ==1  Always=2 |

Table S2. Scoring Protocol for the KAP domain

| **KAP Domain** | **Scored Items** | **Mean score value** | **Cut-off Point** | **Category & Operational Definition** | **Justification** |
| --- | --- | --- | --- | --- | --- |
| **Knowledge** | Continuous mean score of binary items of (*K1-K7*) | 0.14  0.29  0.43  0.57  0.71  0.86  1.00 | 0.43 | Inadequate: ≤ 0.43 Adequate: ≥ 0.43 | Inadequate: 59.4% (n=228) Adequate: 40.6% (n=156)  Sample Median Split: Bypasses absolute threshold limitations due to compressed variations, securing balanced groups for stronger bivariate tests. |
| **Attitude** | Continuous mean score of 5-point Likert scale | 2.00  2.67  3.00  3.33  3.67  4.00  4.33  4.67  5.00 | 3.41 | Un favourable: < 3.41 Favourable: ≥ 3.41 | Modified Bloom’s 70% Cut-off: Implements an absolute benchmark requiring explicit positive agreement trends on the underlying Likert items. Participants scoring < 3.41were classified us having Un favourable Attitude: 10.2% (n=39)  and participants who scoring ≥ 3.41 were classified as having Favourable Attitude 89.8% (n=345) |
| **Practice** | Continuous mean score of practice*(P1-p7)* | 0.00  0.14  0.29  0.43  0.57  0.71  0.86  1.00  1.14  1.29  1.43  1.57  1.71  1.86  2.00 | 1 | Poor:  Good | To facilitate binary logistic regression analysis, the mean score was dichotomized using the scale's absolute mathematical midpoint (1.00) as the cut point.  Participant scoring <1.00were classified as having Poor practice : 50.8% (n=195) and Participant scoring : ≥ 1.00 classified as having Good Practice : 49.2% (n=189) |

Table S3. Description of respondent’s knowledge towards tick and TBD

| **Variables** | **Category** | **Frequency N (%)** |
| --- | --- | --- |
|  |  |  |
| Knowledge | Adequate Knowledge | 228(59.4) |
|  | In adequate Knowledge | 156(40.6) |
| Have you ever seen Ticks? | Yes | 384 (100) |
|  | No | 0(0) |
| If yes, where do you commonly find ticks? | On the animals | 199(51.8) |
|  | In the forests | 80 (20.8) |
|  | On grazing land | 10(2.6) |
|  | All of the above | 95(24.5) |
| In which type of environment do you find ticks most commonly? | Warm environment | 158 (41.1) |
|  | Cold environment | 102 (26.6) |
|  | In Both environment | 124 (32.2) |
| In which seasons of the year do you observe ticks on the cattle’ body most commonly? | Summer | 244 (63.5) |
|  | Winter | 65 (16.9) |
|  | Throughout the year | 75 (19.5) |
| Where do you think the cattle become infested with ticks? | In the forest | 103 (26.8) |
|  | Grazing land | 80 (20.8) |
|  | From the bedding materials | 5 (1.3) |
|  | From fodder grasses | 33 (8.6) |
|  | Don’t know | 163 (42.4) |
| Which type of cattle do you think gets mostly infested by ticks? | Local breeds | 200(52.1) |
|  | Exotic breeds | 126(32.8) |
|  | Both | 25(6.5) |
|  | Don’t know | 33(8.6) |
| Which of the cattle do you think is most affected by tick infestation? | Adult | 62(16.1) |
|  | Heifers | 19(4.9) |
|  | Young calves | 53(13.8) |
|  | Old cattle | 195(50.8) |
|  | Don’t know | 16(4.2) |
|  | All | 39(10.2) |
| Do you think ticks always stay on the body of cattle unless removed? | Yes | 196(51.0%) |
|  | No | 188(49.0%) |
| Have you also seen ticks on the body of other animals? | Yes | 196(71.4%) |
|  | No | 110(28.6%) |
| If yes, in which species of animal did you see? | Small ruminants (sheep and goats) | 19(4.9%) |
|  | Dogs | 78(20.3%) |
|  | Cat and Dogs | 50(13.0%) |
|  | Dogs, Equines, Small ruminants (sheep and goats) | 72(18.8%) |
|  | Bovine(cattle) | 118(30.7%) |
| Do you think cattle can get diseases from ticks? | Yes | 196(51.0%) |
|  | No | 188(49.0%) |
| Have you heard of any tick-borne diseases in cattle? | Yes | 71(18.5%) |
|  | No | 313(81.5%) |
| If yes, where did you hear about the tick-borne diseases in cattle? | Farmers’ training program | 14(19.77%) |
|  | Neighbour | 31(43.7%) |
|  | Friends | 26(36.6%) |
| Have you ever been infested with tick? | Yes | 192(50.0%) |
|  | No | 192(50.0%) |
| Do you think tick cause disease to human? | Yes | 180(46.9%) |
|  | No | 204(53.1%) |

Table S4. Practices of respondents regarding to control of tick infestation

| Variables | Category | Frequency(%) |
| --- | --- | --- |
| The main purpose of rearing cattle in their household | Family consumption of products | 102(26.6%) |
|  | Income through the sale of products | 198(51.6%) |
|  | Income through the sale of animals | 62(16.1%) |
|  | As a source of manure | 22(5.7%) |
| Husbandary practice | All-time free grazing | 54(14.1%) |
|  | A mix of stall feeding and free grazing | 97(25.3%) |
|  | A mix of stall feeding and tethered grazing | 118(30.7%) |
|  | Stall feeding | 115(29.7%) |
| Type of cattle shed they have | Conventional Shed (built with local materials) | 140(36.5%) |
|  | Improved shed with CGI Sheet and concrete flooring | 147(38.3%) |
|  | Improved shed with CGI Sheet and soil flooring | 79(20.6%) |
|  | Open air tethering | 18(4.7%) |
| Frequency of cleaning flooring of their cattle shed | Daily | 265(69.0%) |
|  | Fortnightly | 36(9.4%) |
|  | Never | 13(3.4%) |
|  | Weekly | 70(18.2%) |
| Do you use bedding materials in your cattle shed? | No | 185(48.2%) |
|  | Yes | 199(51.8%) |
| Type of bedding materials They use in cattle shed? | No bedding | 185(48.2%) |
|  | Gravel and san | 98(25.5%) |
|  | Litter leaves | 82(21.4%) |
|  | Straw | 19(4.9%) |
| When do you mostly use bedding materials? | Winter | 161(41.9%) |
|  | Summer | 17(4.4%) |
|  | Throughout the year | 21(5.5%) |
|  | Not at all | 185(48.2%) |
| Which animal health problems are most important to you? | Metabolic diseases, Viral diseases, Bacterial diseases | 23(6.0%) |
|  | Endo-parasites, Tick infestation, Mastitis | 110(28.6%) |
|  | Mastitis, Endo-parasites, Bacterial diseases | 148(38.5%) |
|  | All | 103(26.8%) |
| The main purposes that make them to visit the veterinary centres? | To receive acaricides | 99(25.8%) |
|  | To receive livestock production inputs, To receive deworming drugs, To receive acaricides | 75(19.5%) |
|  | To receive medicines for sick animals, To receive deworming drugs, To receive acaricides | 179(46.6%) |
|  | To seek advice on farming practices | 31(8.1%) |
| Control measures they follow in their farm to address the problem of tick infestation in cattle | Adopt good farm practices | 43(11.2%) |
|  | Injecting ivermectin | 142(37.0%) |
|  | Manually remove the ticks | 32(8.3%) |
|  | Use acaricides | 118(30.7%) |
|  | Use acaricides, Adopt good farm practices | 11(2.9%) |
|  | Use homemade remedies | 38(9.9%) |
| The frequency of applying acaricides on their farm | Weekly | 33(8.6%) |
|  | Fortnightly | 86(22.4%) |
|  | Monthly | 57(14.8%) |
|  | Occasionally | 208(54.2%) |
| Method of applying acaricide they follow | Hand washing | 69(18.0%) |
|  | Injection | 178(46.4%) |
|  | Spraying | 137(35.7%) |
| How long does it take for ticks to fall off from the  Body of cattle after acaricide treatment? | Ticks fall off within a day | 198(51.6%) |
|  | Ticks fall off within a week | 120(31.3%) |
|  | Ticks fall off within few hours | 11(2.9%) |
|  | Don’t Know | 55(14.3%) |
| Method of removing ticks | Collect and burn | 11(2.9%) |
|  | Collect and throw it in the field | 20(5.2%) |
|  | Flush it with running water | 50(13.0%) |
|  | Let it stay on the ground | 251(65.4%) |
|  | Nothing to do | 52(13.5%) |
| What do you do if the veterinary centre has no acaricides? | Apply petroleum and kerosene products | 83(21.6%) |
|  | Apply salt solution | 50(13.0%) |
|  | Brush the animal | 105(27.3%) |
|  | Do nothing | 64(16.7%) |
|  | Manually remove | 82(21.4%) |
| Other than treating tick infestation, where else do you think the acaricides can be used? | Can be used as pesticides in the vegetable fields | 137(35.7%) |
|  | Can be used to get rid of insects, lice, mites and bugs in homes | 112(29.2%) |
|  | Don't know | 135(35.2%) |

Table S5. Frequency distribution of farmers Practices in Acaricide Management and Tick Exposure

| Code | Items | Category | Frequency(%) |
| --- | --- | --- | --- |
| P1 | Do you read the manufacturer's instructions before preparing or mixing the acaricide? | Never | 192(50%) |
|  |  | Sometimes | 110(28.6%) |
|  |  | Always | 82(21.4%) |
| P2 | How do you determine the correct concentration or mixing ratio of the acaricide? | Don’t measure | 167(43.5%) |
|  |  | By estimation | 132(34.4%) |
|  |  | According to instruction | 85(22.1%) |
| P3 | How do you wear protective gear during acaricide handling? | No protection | 159 (41.4%) |
|  |  | Partial gear | 139 (36.2%) |
|  |  | Full gear | 86(22.4%) |
| P4 | How do you apply the acaricide to the cattle? | Wrong method | 139(36.2%) |
|  |  | Partial spraying | 69(18.0%) |
|  |  | Spraying Correctly | 176(45.8%) |
| P5 | Do you observe the recommended withdrawal period for milk and meat after application? | Never | 133(34.6%) |
|  |  | Sometimes | 169(44.0%) |
|  |  | Always | 82(21.4%) |
| P6 | Do you check your body for ticks after handling tick-infested cattle? | Never | 105(27.3%) |
|  |  | Sometimes | 150(39.1%) |
|  |  | Always | 129(33.6%) |
| P7 | Do you check your body for ticks after visiting forests or grazing lands? | Never | 110(28.6%) |
|  |  | Sometimes | 132(34.4%) |
|  |  | Always | 142(37.0%) |
| Practice | | Poor practice | 195(50.8%) |
|  |  | Good practice | 189(49.2%) |
